# Quantum biology in ophthalmology

**DOI:** 10.1038/s41433-024-03245-4

**Published:** 2024-07-23

**Authors:** Ethan Waisberg, Joshua Ong, Mouayad Masalkhi, Andrew G. Lee

**Affiliations:** 1 Department of Clinical Neurosciences, University of Cambridge, Cambridge, United Kingdom; 2 Michigan Medicine, University of Michigan, Ann Arbor, MI USA; 3 University College Dublin School of Medicine, Belfield, Dublin, Ireland; 4 Center for Space Medicine, Baylor College of Medicine, Houston, TX USA; 5 Department of Ophthalmology, Blanton Eye Institute, Houston Methodist Hospital, Houston, TX USA; 6 The Houston Methodist Research Institute, Houston Methodist Hospital, Houston, TX USA; 7 Departments of Ophthalmology, Neurology, and Neurosurgery, Weill Cornell Medicine, New York, NY USA; 8 Department of Ophthalmology, University of Texas Medical Branch, Galveston, TX USA; 9 University of Texas MD Anderson Cancer Center, Houston, TX USA; 10 Texas A&M College of Medicine, Bryan, TX USA

**Keywords:** Scientific community, Physiology

## Introduction

Quantum biology is the application of quantum theory to describe aspects of biology that are insufficiently described by classical laws of physics. This field involves the study of how light interacts with biological systems. Prior to the 20th century, physics and biology rarely intersected and biological systems were thought to be too complex to be represented using mathematical methods [1]. However, in present-day ophthalmology, the use of physics is essential, from the use of lasers to correct errors in refraction [2], to using a stream of air to measure intra-ocular pressure [3]. The visual pathway is fundamentally quantum mechanical in nature, involving electron transfer and energy transfer based on surface hopping [4]. Currently, the usage of quantum biology in ophthalmology remains an alien concept to most ophthalmologists and ophthalmic researchers. In the same way that emerging technologies such as virtual reality (VR) [5–7] and artificial intelligence (AI) [8–10] were rapidly integrated into ophthalmology to benefit patients, quantum biology can be a key future area of research to provide novel diagnostics and treatments. Further research in quantum biology can potentially lead to the development of non-invasive ophthalmic therapeutic devices and new drugs to heal the eye.

Since the early 20th century, the field of quantum biology has evolved significantly and quantum phenomena such as quantum coherence and tunnelling are now widely accepted as being involved in vital processes for enzyme action and energy transfer for every living cell [11]. Recent evidence has shown that light-harvesting systems such as photoreceptors, have evolved quantum features including tunnelling, coherence, and entanglement to function at remarkable levels of efficiency [12]. Tunnelling is a quantum mechanical phenomenon where particles such as electrons or protons travel through a potential barrier that they should not be able to pass [13]. In biological systems, quantum tunnelling is vital for processes that include enzyme reactions and photosynthesis [13]. It allows electrons or protons to pass through energy barriers and thus significantly enhances the speed and efficiency of these reactions [13]. This capability is fundamental to the effective functioning of cellular processes at the atomic level and contributes to the high efficiency observed in biological systems such as light-harvesting complexes in photosynthesis [13] (Table 1).

**Table 1:** Key quantum phenomena and definitions.

Quantum Phenomena	Definition
Tunnelling	Quantum tunnelling is a phenomenon where particles travel through a barrier that classical physics would deem impossible [16]. This occurs when a particle, such as an electron, passes through an energy barrier despite not having enough energy to overcome it [16]. Tunnelling is vital in various biological and technological processes, such as enzymatic reactions and semiconductor physics [16].
Coherence	Quantum coherence refers to the condition where particles like electrons or photons maintain a fixed phase relationship [16]. This principle is essential in quantum computing as it enables the creation and manipulation of quantum bits to perform computations that are exponentially faster than classical computers [16].
Entanglement	Quantum entanglement is a phenomenon where pairs or groups of particles interact such that the quantum state of each particle cannot be delineated independently of the state of the other particles, even when the particles are separated by large distances [16]. This inseparable relationship enables phenomena such as faster-than-light communication of information between entangled particles [11, 16].

Lyu et al. [14] focused on the role of melanin in the turnover of photoreceptor discs in the retinal pigment epithelium (RPE) and its role in age-related macular degeneration (AMD) and Stargardt disease. Investigators utilized “chemiexcitation,” which is a quantum chemistry phenomenon where a chemical reaction leads to the formation of electronically excited states [15]. This process does not involve light energy but instead electrons are triggered by molecular interactions and energy transfers within chemical structures, e.g. the decomposition of dioxetanes [14]. This phenomenon can cause non-radiative energy transfer to DNA or other biomolecules, leading to effects such as bioluminescence or melanin-mediated transfer of energy [14]. In this investigation chemiexcitation of electrons significantly impacts the degradation of lipofuscin—a pigment that accumulates due to the breakdown of photoreceptor discs and is involved in retinal degeneration [14].

The investigation employed high-resolution electron microscopy, genetics, pharmacological treatments, and chemical interventions to demonstrate the degradation pathways of lipofuscin in both pigmented and albino Abca4-/- mice. It was found that in pigmented mice, chemiexcitation aided by melanin effectively reduced lipofuscin levels [14]. On the other hand, albino mice, which lack melanin, showed an accumulation of lipofuscin and related structures unless treated directly with a synthetic dioxetane that induces chemiexcitation (thus bypassing the need for melanin) [14]. Results highlighted that melanin not only aids in the physical breakdown of photoreceptor disc remnants but also chemically through electron excitation, which is pivotal for mitigating lipofuscin buildup and therefore, could be potentially used in delaying the progression of AMD and Stargardt disease [14].

Living systems have had 3.5 billion years of evolutionary optimization, and during this vast time span, these biological systems have likely developed the ability to manipulate quantum dynamics in ways that are not yet fully understood [11]. Advancements in single particle imaging, ultrafast spectroscopy and time-resolved microscopy, and other similar experimental techniques will further enable the study of ophthalmic systems at small scales, and will further reveal the interplay between classical physics and quantum effects.

Future research could also examine if any distinct quantum signatures can be seen in a variety of retinal pathology [12]. As quantum processes in the eye are better understood, this information may also provide novel insights that can be transferred to the field of quantum computing, medical imaging and the development of novel therapeutics. Further multidisciplinary research involving ophthalmologists, spectroscopists, chemists and theoretical physicists to examine this area further.

## References

[1] Lambert N, Chen YN, Cheng YC, Li CM, Chen GY, Nori F. Quantum biology. Nat Phys. 2013;9:10–8.

[2] Ong J, Waisberg E, Masalkhi M, Suh A, Kamran SA, Paladugu P, et al. “Spaceflight-to-Eye Clinic”: Terrestrial advances in ophthalmic healthcare delivery from space-based innovations. Life Sci Space Res. 2024;41:100–9. 10.1016/j.lssr.2024.02.003 38670636

[3] Waisberg, E J Ong, M Masalkhi, P Paladugu, AG Lee, J Berdahl Precisional modulation of translaminar pressure gradients for ophthalmic diseases. Eur J Ophthalmol 11206721231199779 (2023) 10.1177/11206721231199779. 10.1177/11206721231199779 PMC11408950 37670516

[4] Marais A, Adams B, Ringsmuth AK, Ferretti M, Gruber JM, Hendrikx R, et al. The future of quantum biology. J R Soc Interface 2018;15:20180640. 30429265 10.1098/rsif.2018.0640 PMC6283985

[5] Zaman N, Ong J, Waisberg E, Masalkhi M, Lee AG, Tavakkoli A, et al. Advanced visualization engineering for vision disorders: a clinically focused guide to current technology and future applications. Ann Biomed Eng. 2023;52:178–207. 10.1007/s10439-023-03379-8. 37861913 10.1007/s10439-023-03379-8

[6] Brent Woodland M. et al. Applications of extended reality in spaceflight for human health and performance. *Acta Astronautica* S0094576523005945 (2023) 10.1016/j.actaastro.2023.11.025.

[7] Waisberg E, Ong J, Masalkhi M, Zaman N, Sarker P, Lee AG, et al. Apple Vision Pro: the future of surgery with advances in virtual and augmented reality. Ir J Med Sci. 2023;193:345–6. 10.1007/s11845-023-03457-9. 37422552 10.1007/s11845-023-03457-9

[8] Masalkhi M, Ong J, Waisberg E, Zaman N, Sarker P, Lee AG, et al. Large language models for post-operative guidance in refractive surgery. AME Surg J. 2024;4:2–2.

[9] Paladugu PS, Ong J, Nelson N, Kamran SA, Waisberg E, Zaman N, et al. Generative adversarial networks in medicine: important considerations for this emerging innovation in artificial intelligence. Ann Biomed Eng. 2023;51:2130–42. 10.1007/s10439-023-03304-z. 37488468 10.1007/s10439-023-03304-z

[10] Suh A, Ong J, Kamran SA, Waisberg E, Paladugu P, Zaman N, et al. Retina oculomics in neurodegenerative disease. Ann Biomed Eng. 2023;51:2708–21. 37855949 10.1007/s10439-023-03365-0

[11] McFadden J, Al-Khalili J. The origins of quantum biology. Proc R Soc A 2018;474:20180674. 30602940 10.1098/rspa.2018.0674 PMC6304024

[12] Sia PI, Luiten AN, Stace TM, Wood JP, Casson RJ. Quantum biology of the retina. Clin Exp Ophthalmol. 2014;42:582–9. 24979043 10.1111/ceo.12373

[13] Trixler F. Quantum tunnelling to the origin and evolution of life. Curr Org Chem. 2013;17:1758–70. 24039543 10.2174/13852728113179990083 PMC3768233

[14] Lyu Y, Tschulakow AV, Wang K, Brash DE, Schraermeyer U. Chemiexcitation and melanin in photoreceptor disc turnover and prevention of macular degeneration. Proc Natl Acad Sci USA. 2023;120:e2216935120. 37155898 10.1073/pnas.2216935120 PMC10194005

[15] Ueda K, Zhao J, Kim HJ, Sparrow JR. Photodegradation of retinal bisretinoids in mouse models and implications for macular degeneration. Proc Natl Acad Sci USA. 2016;113:6904–9. 27274068 10.1073/pnas.1524774113 PMC4922174

[16] Matarèse BFE, Rusin A, Seymour C, Mothersill C. Quantum biology and the potential role of entanglement and tunneling in non-targeted effects of ionizing radiation: a review and proposed model. Int J Mol Sci. 2023;24:16464. 38003655 10.3390/ijms242216464 PMC10671017

